# Childhood Experiences of Companion Animal Abuse and its Co-Occurrence with Domestic Abuse: Evidence from a National Youth Survey in Norway

**DOI:** 10.1177/08862605211072176

**Published:** 2022-02-12

**Authors:** Karianne Muri, Else-Marie Augusti, Margunn Bjørnholt, Gertrud Sofie Hafstad

**Affiliations:** 1 Faculty of Veterinary Medicine, Norwegian University of Life Sciences, Ås, Norway; 2 25566 Norwegian Centre for Violence and Traumatic Stress Studies, Oslo, Norway; 3 Department of Sociology, University of Bergen, Bergen, Norway

**Keywords:** animal abuse, child abuse, coercive control, domestic abuse, physical abuse, psychological abuse, violence

## Abstract

It is increasingly acknowledged that companion animal abuse often occurs in the same contexts as other types of abuse, particularly domestic abuse. However, the co-occurrence and strengths of these associations in the general population have not been well established in research. With data from a large representative sample of Norwegian adolescents, we aimed to determine 1) the extent to which Norwegian children are exposed to companion animal abuse in the family, 2) whether and how companion animal abuse is linked to other forms of domestic abuse that children experience, and 3) background factors associated with companion animal abuse. A total of 9240 adolescents aged 12–16 years (*M*_age_ 14.7) participated in the digital school-based survey. Four percent (*n* = 380) reported that they had ever witnessed a parent being violent towards a family companion animal, whereas 1% (*n* = 125) had experienced that an adult in the household had threatened to harm a companion animal. There was a substantial overlap between companion animal abuse and child abuse, and it most frequently co-occurred with psychological abuse and less severe forms of physical child abuse. This resonates with conceptualizations of domestic abuse as an ongoing pattern of psychological abuse and coercive control. The risk factors identified for companion animal abuse in this representative sample of adolescents were similar to known risk factors for domestic abuse. Low socioeconomic status and parents’ substance abuse, parents’ psychiatric illness, and parents’ history of incarceration entailed a greater risk of experiencing companion animal abuse. We conclude that companion animal abuse co-occurs with other forms of domestic abuse and that it may be considered a part of the repertoire of domestic abuse that impacts children.

It is increasingly acknowledged that animal abuse often occurs in the same contexts as other types of abuse, particularly domestic abuse. This reported association between animal abuse and domestic abuse, including child abuse, has been termed “the link” (Jegatheesan et al., 2020). Despite an increasing focus on the many facets of “the link” over recent years, the co-occurrence and strengths of associations in the general population is still not well substantiated in research. Unfortunately, animal abuse is not routinely integrated in survey studies of domestic abuse, hence, apart from one recent study (Fitzgerald et al., 2020), there is a dearth of large-scale, representative studies of animal abuse and its link to domestic abuse. Most studies exploring associations between animal abuse and domestic abuse are based on small samples and a limited scope, often drawing on clinical populations (e.g., Ascione et al., 1997; DeViney et al., 1983; Fitzgerald et al., 2019; Volant et al., 2008). Other methodological issues in previous research have been the reliance on informants’ self-report of own violent behavior, their report of their children’s behavior or experiences, and/or on memory of incidents in the distant past (e.g., DeGue & DiLillo, 2009; Rosenbaum & O’Leary, 1981; Volant et al., 2008).

This paper presents a large-scale study from a general population of adolescents in Norway, and our aim is to contribute towards reducing the described knowledge gap. To our knowledge, this is the first large-scale study, nationally or internationally, mapping exposure to animal abuse and its co-occurrence with child abuse and other forms of domestic abuse, as experienced by a general population of adolescents.

## The Many Facets of “The Link”

Over the last couple of decades, the links between domestic abuse and animal abuse have been increasingly studied within social sciences (e.g., Ascione et al., 2007; Volant et al., 2008). Harming companion animals, or threats to do so, have long been recognized as ways to coerce, intimidate, and manipulate domestic abuse victims (Alleyne & Parfitt, 2019; Randour et al., 2019). Victims of domestic abuse frequently remain in abusive relationships because of concerns for the safety of their companion animals if left with the offender (Ascione, 2007). Several other ways in which animal cruelty is linked to domestic abuse has been described by Roguski (2012). Perpetrators may threaten to harm and/or actually harm companion animals as a demonstration of anger or jealousy (e.g., of a child’s emotional bond with a companion animal), or as a mechanism to punish family members. Sometimes, the violence against the companion animal is “collateral,” that is, a secondary consequence of the perpetrator’s behavior. Children can also be forced to harm animals themselves, or to engage in sexual activities with them, as part of the domestic child abuse (Roguski, 2012).

One frequently cited article, based on a sample of 860 American college students, described associations between violence against children, intimate partners, and animals (DeGue & DiLillo, 2009). About 60% of the students who had experienced animal abuse, either as an offender or as a witness, had also experienced child abuse or other forms of domestic abuse. Conversely, about 30% of the students with experiences of child abuse or other forms of domestic abuse had also been exposed to animal abuse. In other words, the presence of animal abuse was a stronger marker of domestic abuse than vice versa. It was proposed that animal abuse perpetrated by parents or children therefore may serve as a red flag for the presence of child abuse or other forms of domestic abuse. Studies based on more limited samples, include an older American study of families with documented child abuse, among which animals had been abused by family members in 60% of the families (DeViney et al., 1983). In another American study, more than 70% of female victims of domestic abuse staying at a shelter reported that the violent offender also had harmed, killed, or threatened to harm animals, and that the woman and/or her children had witnessed more than 75% of the animal abuse incidents (Ascione et al., 1997). Similar interrelationships have also been reported in more recent studies (e.g., Bright et al., 2018; Simmons & Lehmann, 2007; Volant et al., 2008).

Recently, Fitzgerald et al. (2020) published data on the associations between animal abuse and emotional and financial forms of intimate partner violence, based on a large-scale and representative cross-sectional survey in Canada. In their study, threatened or enacted violence against companion animals increased the probability of all the measured forms of emotional and financial intimate partner violence. Their study, as the first of its scale, provided more empirical support to the links between animal abuse and intimate partner abuse in an adult population (above 15 years of age). However, the publication does not report the overall prevalence of animal abuse reported by the respondents and has delimited its focus to emotional and financial domestic abuse, while excluding physical abuse. To our knowledge, no similar large-scale study exists on the links between animal abuse and child abuse.

The present study was conducted in Norway, a country with almost 5.4 million inhabitants and a high GDP per capita (67,326 U.S. dollars in 2020, (Statista, 2021). The country is defined as a social democratic welfare state (Esping-Andersen, 1990). It has a welfare model rooted in egalitarian ideals, characterized by a comprehensive social security system, universal health care, and redistribution of wealth through taxation. Public education is cost free from primary school to higher education. About one-third of the households in Norway have companion animals (Norwegian Ministry of Agriculture, 2002), mostly dogs or cats, and more than half of these households constitute families with children. In other words, many Norwegian children grow up with at least one companion animal.

### Potential Consequences of “The Link” for Children

In many families, the companion animal is an integral part of the family life and is treated as a cherished family member. For a child who is exposed to abuse at home, the emotional bond to a companion animal may serve as an important source of comfort and support. The animal can provide the child with consolation, reduce feelings of loneliness, and have other psychological benefits (Andreassen et al., 2013; McConnell et al., 2011). The human–animal bond may even have a protective effect against suicidality in domestic abuse victims (Fitzgerald, 2007). It is, however, important to be aware that this emotional bond is something that can be exploited by an offender, who may manipulate the child to silence or to do as the offender wishes by harming or threatening to harm the animal. Witnessing animal abuse as a child is a form of psychological abuse that may be traumatizing and harmful to a child’s development (Boat, 2014; Randour et al., 2019). Thus, the harm and suffering that animal abuse inflicts on the animal victims themselves may extend to the child witnessing it. Qualitative research has indeed described how children who are exposed to animal abuse at home may intervene in an attempt to protect their companion animal, by pleading the offender to leave the animal alone or physically going between the animal and the offender (McDonald et al., 2015). In a study of abused mothers in a shelter in the US, more than 50% of their children reported that they had tried to protect their animal in this way (Ascione et al., 1997). One of the few studies with data on the prevalence of parent-perpetuated animal abuse in a general sample of children, is a study investigating associations between exposure to domestic violence and self-reported animal abuse among almost 1400 adolescents (9–17 years of age) from Rome (Baldry, 2003). Nine percent of the adolescents reported that their father had harmed an animal, while 5.1% reported that their mother had harmed an animal.

Due to the described facets of “the links,” some have advocated for routine cross-reporting between child welfare agencies and animal welfare authorities. However, some researchers have raised concerns that cross-reporting may be introduced based on weak or lacking empirical evidence of these associations among the general population (Beirne, 2009; DeGue & DiLillo, 2009; Patterson-Kane & Piper, 2009). With our study we wish to fill some of the knowledge gaps about “the links” by presenting empirical data on the extent of animal abuse and its co-occurrence with child abuse, based on a large-scale survey of adolescents in Norway. The aim of this study was therefore to determine 1) the extent to which Norwegian children are exposed to animal abuse within the family, 2) whether and how companion animal abuse is linked to other forms of domestic abuse that children experience, and 3) background factors associated with companion animal abuse.

## Method

### Sample

The present study is based on results from the first national survey on child abuse and neglect among a representative sample of Norwegian 12 to 16-year-olds (Hafstad et al., 2020). A total of 9240 adolescents participated in the study, corresponding to a 75.5% response rate. Mean age was 14 years, and boys and girls were equally represented (less than 1% did not identify as gender binary). The majority of the respondents were Norwegian and had parents born in Norway, but almost 20% were born abroad or had two parents born abroad. Almost 80% of the adolescents lived with both parents, who at the time of the survey lived together. For the distribution of other demographic variables, see Table 1.

**Table 1.** Distribution of the Sample, with Frequency (*n*) and Prevalence (%) of Companion Animal Abuse Reported by Each Subgroup, and *p* Values from Chi-Square Tests.

		Total *n*	*n*	%	*p* value
Gender					
	Boy	4484	136	3.0	
	Girl	4559	235	5.2	<.001
Country of origin					
	Parents of immigrant background	2286	72	3.1	
	Parents without immigrant background	6617	293	4.4	.08
Living arrangement					
	Living with both parents	6360	217	3.4	
	Living with one of the parents	2667	154	5.8	<.001
Family affluence					
	Low	331	43	13.0	
	High	8637	323	3.7	<.001
Parental risk factors					
	High risk*	1697	151	9.4	
	Low risk	7396	218	2.9	<.001

^a^Parental risk factors are defined as living with parents who had either a mental illness, alcohol or drug use problems, or who have ever been in prison.

### Procedure

The study had a cross-sectional design and participants were recruited from schools. The vast majority of schools in Norway are public, as reflected in the sample. The survey was conducted during school hours, and data collection took place during the months of January and February 2019. A web-based survey was administered on PCs or tablets in the classroom. A digital survey format allows for flexibility in follow-up questions, and specifically tailored questions for respondents yielding experiences of child abuse and neglect. We made use of behavior specific questions which is the recommended survey methodology, also in research on child abuse and neglect. The adolescents provided independent consent, without parental consent.

### Background Variables

The participating youth were asked about their age, gender, and place of birth (in Norway or in another country), with the two latter treated as dichotomous variables, and age as a continuous variable. To determine parents’ country of origin, participants were asked to indicate whether their mother and father, respectively, were born in Norway or a Nordic country (0), a European country (1), or a country outside Europe (2). Responses to these questions were combined into a composite variable on parents’ country of origin (both parents born either in Norway or the Nordic countries (0), at least one parent born in a European country other than a Nordic country (1), or at least one parent born in a country outside of Europe (2). Perceived family affluence was reported as 1) whether the adolescent experienced the family as having sufficient economic means to buy necessary goods or 2) whether the adolescent had to decline after-school activities due to family finances. The first question was rated on a 4-point scale from 0 = *completely agree* to 3 = *completely disagree*, and the latter was rated on a 4-point scale from 0 = *never* to 3 = *often*. A dichotomous composite score of perceived family affluence was generated based on responses to these two questions; if category 2 or 3 were indicated on either of the two questions, a low perceived family affluence score (1) was allocated to that individual. Parents’ problems related to mental health, alcohol or drug misuse, or incarceration were measured on a 3-point scale (0 = *no*, 1 = *yes*, or 2 = *unsure*). In the present study, this variable was dichotomized, reporting only adolescents yielding *no* or *yes* to this question, and treating the *unsure* category as missing. Adolescents were also asked to indicate whether they resided with both parents at the time of participation, or if they had experienced any type of family disruption and therefore were not living with one or both of their parents. The variable was dichotomized as 0 = *residing with both parents* and 1 = *living in a split family*.

### Violence and Abuse Measures

We used six questions mapping physical abuse from the Parent-Child Conflicts Tactics Scale (PCCTS; Straus et al., 1998). The questions were modified from a Norwegian (Myhre et al., 2015) and a Swedish (Jernbro & Janson, 2016) prevalence study regarding the same topic. The questions ranged from less severe physical abuse, such as pinching, tugging hair, shaking/pushing hard, or slapping, to the more severe forms, such as punching/hitting with object, kicking, or beating up. The respondents were asked to report the frequency with which during their childhood they had experienced an adult at home enact each item, using a 4-point scale from 0 = *never* to 3 = *often*.

Eight questions pertaining to different types of psychological abuse were included. These questions were also inspired by items in the PCCTS (Straus et al., 1998). The items included experiences of being ridiculed, parents threatening to leave or send the child away, threats of being hit or physically hurt, being locked inside or outside the home, and threats of harming a companion animal. The respondents were asked to report the frequency with which they had experienced these different forms of psychological abuse by an adult at home during their childhood, using a 4-point scale (from 0 = *never* to 3 = *often*). Psychological abuse was defined as having experienced at least two or more types of psychological abuse, or more than one incidence of one type of psychological abuse. We define it as psychological abuse whenever there is a pattern of abuse, i.e., that it happens repeatedly (scored as *sometimes* or *often*).

Six items were included to assess sexual abuse by an adult offender. The questions were inspired by similar questions in a study of adolescents’ experiences of violence and sexual abuse in Norway (Mossige & Stefansen, 2007), but adapted to the age of the target group of the present study. To assess domestic abuse, six items about the adolescents’ experiences (seen or heard) of violence between caretakers in the home were included. Questions were adapted from a Norwegian study on older adolescents’ violence and abuse experiences (Mossige & Stefansen, 2016). Finally, two questions were asked regarding witnessing siblings or companion animals, respectively, being intentionally hit or hurt by an adult member of the household. Both items were dichotomous (*yes* or *no*).

### Ethical Considerations

To ensure adolescents’ informed consent, several measures were taken to make sure that the information given prior to participation was understandable, relevant, age appropriate, and as coherent as possible across different schools and classrooms. A 5-minute animation film was developed to meet the ethical standards for informed consent in a youth population. The film was shown to all invited adolescents prior to consent, and contained information about the purpose of the study, the participants’ rights (including the right to decline participation or to withdraw at a later time), as well as information about the web-based survey format.

Due to the young age of the participants, as well as the sensitivity of the themes covered in the survey, a careful follow-up plan was developed. To meet the adolescents’ potential needs for follow-up, a contact form was generated, allowing all invited school pupils to respond if they wanted to be contacted by a professional helper after the survey. A total of 480 individuals, 5% of all invited youth, made use of this invitation.

The study protocol has been approved by the Regional Committee for Ethics in Medical and Health research in the South-eastern region of Norway (Case # 2018/522).

### Data Management and Statistical Analyses

Results are largely presented as frequency (*n*) and percentage (%) of the sample. We also report means (*M*) and standard deviations (*SD*) for continuous variables. A chi-square (χ^2^) test was used for comparison of categorical variables. To investigate interrelationships, we used logistic regression analyses, and results are reported as odds ratios (*OR*). All analyses were conducted in IBM SPSS Statistics for Windows, version 26 (IBM Corp., Armonk, NY, USA).

## Results

### Experience With Animal Abuse

A total of 9240 adolescents aged 12–16 years returned the survey, of which 4% (*n* = 380) reported that they had witnessed a parent being violent towards a family companion animal, whereas 1% (*n* = 125) had experienced that an adult in the household had threatened to harm a companion animal. Table 1 presents the prevalence of companion animal abuse within different subgroups of the sample. Overall, girls reported animal abuse exposure more often than boys did. Other groups who reported exposure to animal abuse more frequently were adolescents from single-parent households, adolescents living in families with financial hardship, adolescents whose parents have ever had mental health problems, drug use problems, or who had been incarcerated.

### Co-Occurrence of Other Forms of Abuse

To investigate the possible co-occurrence of animal and child abuse, we ran a set of chi-square tests comparing the experiences of physical, psychological, and sexual abuse in the group reporting companion animal abuse and the group without companion animal abuse exposure, respectively. Overall, own abuse experiences were grossly overrepresented in the group of adolescents who had experienced companion animal abuse, as compared to those who did not report animal abuse (see Table 2). Among the adolescents with animal abuse experiences, 18.8% had also experienced physical abuse, such as being beaten up, or hit with an object or a fist, as compared to 3.4% in the non-animal abuse group. An even higher proportion, 56.8%, had experienced the less severe forms of physical abuse, such as hairpulling, pinching, or being slapped, as compared 17.1% in the non-animal abuse group.

**Table 2.** Frequency (*n*) and Percentage (%) of Overlap Between Different Forms of Child Abuse and Exposure to Companion Animal Abuse.

Form of Child Abuse	Total *n*	CA Abuse		No CA Abuse	
		*n*	%	*n*	%
Being hit with fist or hard object, kicked, or beaten up	369	71	18.8	298	3.4
Being slapped pinched, or pulled by the hair	1703	216	56.8	1487	17.1
Psychological abuse	2970	293	77.3	2677	30.9
Seeing siblings being hit	653	109	28.7	544	6.2
Sexual abuse	534	81	21.5	453	5.2

*Note*. CA = Companion Animal.

About three quarters (77.3%) of animal abuse exposed adolescents had experienced psychological abuse from their parents, including having been repeatedly humiliated, ridiculed, belittled, or threatened. Finally, about one-third (28.7%) of the adolescents with animal abuse experiences had also witnessed their sibling(s) being abused, whereas the corresponding proportion for the non-animal abuse group was 6.2%. In all, psychological abuse and less severe physical abuse most frequently co-occurred with companion animal abuse, whereas severe physical abuse and sexual abuse were less strongly associated with companion animal abuse.

### Risk Factors for Companion Animal Abuse

We were particularly interested in groups with a higher risk of experiencing companion animal abuse, and therefore ran a logistic a regression model investigating a set of predefined risk factors for companion animal abuse. These factors were defined primarily based on their established status as putative risk factors for child abuse.

As can be seen in Table 3, psychological abuse was strongly related to companion animal abuse; having experienced psychological abuse at home was associated with 4-fold odds of experiencing companion animal abuse. Physical abuse, such as hairpulling, pinching or slapping, was associated with more than doubled odds for experiencing companion animal abuse. These less severe forms of physical abuse co-occurred more often with companion animal abuse than did the more severe types of physical abuse, such as being beaten up or hit with a fist or an object. Additionally, risk factors such as low socioeconomic status and parents’ substance abuse, psychiatric illness, and history of incarceration, entailed a greater risk of experiencing companion animal abuse, whereas being of immigrant background was associated with lower risk of companion animal abuse. Although our data are not suited to confirm this, it should be noted that families of immigrant background are thought to have companion animals to a lesser extent than majority Norwegian families.

**Table 3.** Associations Between Companion Animal Abuse and Forms of Child Abuse, Controlling for Other Family Risk Factors.

	*OR*	95% *CI*
Physical abuse (slapping, hairpulling, pinching)	2.52	1.95–3.28
Severe physical abuse (hitting with object, kicking, beating)	1.54	1.10–2.17
Psychological abuse	4.03	3.03–5.39
Sexual abuse	1.83	1.36–2.49
Immigrant status (ref. Non-immigrant)	0.44	0.30–0.63
Family affluence (ref. High family affluence)	1.56	1.06–2.30
Parent risk factors (mental illness, drugs, incarceration)	1.56	1.22–1.99

*Note.* Dependent Variable: Companion Animal Abuse; *OR* = Odds Ratios; *CI* = Confidence Interval.

To investigate the hypothesis that animal abuse is a potential marker of child abuse, we ran a second set of logistic regression models in which child physical abuse (of any severity) was included as the dependent variable, and companion animal abuse served as an independent variable, while controlling for other risk factors. Having witnessed companion animal abuse was associated with more than twofold odds for experiencing physical abuse in the home; *OR* 2.68, (95% *CI* 2.10–3.45), *p* < .001, also when controlling for background variables as well as psychological and sexual abuse. These analyses do indeed show a similar pattern as the former ones: when controlling for other forms of child abuse and family risk factors, companion animal abuse is still associated with increased odds of experiencing physical abuse in the home.

## Discussion

Being the first large-scale youth study assessing both companion animal and child abuse, this study fills a lacuna in the research on the presumed links between animal abuse and domestic abuse.

Among the responding adolescents in the present study, 4% reported that they had experienced that an adult at home had harmed a companion animal. This is the only study of this kind with a large representative sample, and smaller studies with child samples published in the past (e.g., Baldry, 2003) are not directly comparable due to methodological differences. We found that more girls than boys had observed animal abuse. This may reflect the larger general exposure of girls in the study. More girls had experienced multiple forms of violence, and in particular, girls were exposed to more psychological violence. The latter is notable, as we found a strong link between exposure to animal abuse and psychological violence.

Among the adolescents in our study, 1% had experienced that an adult had threatened to harm a companion animal. It has been assumed that threatening to harm companion animals is more prevalent than actual physical harm to them (McPhedran, 2009). In line with this assumption is a study comparing the prevalence of partner-perpetuated animal abuse experienced by women in shelters versus women in a community sample (Ascione et al., 2007). In the community sample, the prevalence of threats to harm a companion animal (12.5%) was more than twice the prevalence of actual harm to pets (5%). The low prevalence of threats of animal abuse in the present study—much lower than the prevalence of enacted animal abuse reported—was therefore not in line with our expectations. However, lower rates of threats of animal abuse compared to actual harm of animals have been reported in samples from clinical populations, for example, in the shelter sample of the abovementioned study by Ascione et al. (2007) and in domestic abuse programs (Hartman et al., 2018). It is not known how well the concept of threats has been defined and explained to the informants across these studies. In our study, the question was asked as part of the mapping of psychological abuse and separate from the question about witnessing animal abuse. Whether this may have affected the adolescents’ understanding of the question, is unclear.

Our study found a clear association between witnessing an adult harming a companion animal and experiences of other forms of domestic abuse, particularly being subjected to psychological abuse and less severe forms of physical abuse. More than three quarters of the adolescents exposed to animal abuse were themselves victims of psychological abuse, and more than half of them had been subjected to less severe forms of physical abuse. Our results thus provide support to previous research findings about the co-occurrence of animal abuse and psychological abuse. Childhood psychological abuse was the only type of domestic abuse that was a significant marker for animal abuse experiences among American college students (DeGue & DiLillo, 2009). This co-occurrence of animal abuse and psychological abuse is also in concordance with the pattern recently reported in connection to intimate partner abuse (Fitzgerald et al., 2020). Experiencing at least one form of psychological abuse was significantly more common among women who had experienced companion animal abuse than among those who had not experienced animal abuse (Fitzgerald et al., 2020).

Witnessing animal abuse and being a direct victim of domestic abuse were approximately equally strong markers of one another in the present study, as opposed to the study of American college students, in which animal abuse was a stronger marker of domestic abuse than vice versa (DeGue & DiLillo, 2009). However, the strengths of associations vary among the different types of domestic abuse. Our study indicates that exposure to psychological abuse and less severe forms of physical abuse to a larger degree increases the probability of co-occurring animal abuse than severe physical abuse does. The more severe forms of physical abuse, sexual abuse, or witnessing a sibling being abused also co-occurred with animal abuse, but to a lesser degree. This is contrary to some previous studies on adult populations that have indicated that animal abuse more commonly co-occurs with severe forms of domestic abuse (Ascione, 2007). Hence, rather than mainly co-occurring with a type of abuse that is severe but nevertheless infrequent, animal abuse most commonly co-occurs with the most prevalent types of child abuse in the present sample. This is important to note from an applied perspective. The high co-occurrence of animal abuse with psychological and less severe physical abuse also resonates with theorizations that center on psychological abuse and coercive control as the core elements of domestic abuse, most poignantly formulated by Evan Stark (Stark, 2009), as well as Michael Johnson’s (1995) related concept of patriarchal/intimate partner terrorism. Likewise, child abuse is also increasingly conceptualized through the lens of coercive control and psychological abuse (Katz et al., 2020; Øverlien, 2013).

Socioeconomic and demographic risk factors are well known and documented in the child abuse literature (Patwardhan et al., 2017; van Ijzendoorn et al., 2020). The present study confirms that children’s animal abuse experiences are associated with similar risk factors, and the more risk factors present, the higher the likelihood of animal abuse exposure. Low socioeconomic status, parental substance abuse, psychiatric illness, or incarceration were all associated with animal abuse in this representative sample. This is in accordance with the recent study from Canada (Fitzgerald et al., 2020), in which lower levels of income were associated with a higher likelihood that the participating women reported exposure to companion animal abuse. However, comparisons are difficult, as socioeconomic status is not uniformly measured across studies. That said, the risk factors identified in the present study generally echo the findings from studies on child abuse and neglect (Patwardhan et al., 2017; van Ijzendoorn et al., 2020).

Our data revealed a lower risk of animal abuse in families with parents with an immigrant background. A possible explanation may be that different cultural backgrounds also entail different attitudes towards animals. Hartman et al. (2018) suggested that if animals to a lesser degree are considered members of the family, they may be less likely to be harmed as a form of coercion, but the cause of the lower risk in our sample is mere speculation.

### Strengths and Limitations

Due to the large and representative sample, this study presents unique data on the prevalence of companion animal abuse and its co-occurrence with other forms of domestic abuse, as reported by 12 to 16-year-old children in Norway. It demonstrates that more knowledge about animal abuse can add to the understanding of domestic abuse as a phenomenon, and in that respect, highlighting the role of animal abuse as a component of psychological abuse and coercive control. Despite its merits, the study also has some limitations. Our data only includes the abuse enacted by adult family members and does not distinguish between animal abuse intended to control or threaten family members, and animal abuse intended to control or punish the animal itself. Nor was any distinction made between different severities and forms of animal abuse, that is, physical, psychological, or sexual abuse. Rough training methods of animals, particularly of dogs, may not be uncommon, and may or may not have been reported as abuse by the participating adolescents.

Through the thorough recruitment process, we obtained a sample that was representative of the population of 12 to 16-year-old school pupils in Norway. Therefore, the sample is likely to also represent the diversity of human differences within this age group, in terms of, for example, ethnicity, nationality, socioeconomic status, religion, gender, and sexual orientation. Because of the scarcity of large-scale studies of this kind, our overriding aim was to obtain a broad picture of the links between animal abuse and child abuse, whereas an in-depth exploration of the role of human differences was outside the scope of this study. Thus, we have only briefly considered certain diversity issues, with focus on gender, country of origin, socioeconomic status, and parental risk factors.

Whether our findings are representative for other countries is uncertain. There may be differences both in domestic abuse and animal abuse rates between countries, due to, for example, cultural and socioeconomic factors. Norway has a long egalitarian tradition of democratizing relations in the family and society, and a zero tolerance of violence. This includes a legal ban on physical discipline of children, as well as a strict animal welfare legislation with mandatory reporting of animal abuse.

### Practical Implications

Recognizing the possibility of animal abuse in the context of domestic abuse adds to our understanding of family violence. The present study thus expands the methodological and theoretical understanding of companion animal abuse, and it may contribute to the ongoing controversy regarding how to measure and theorize domestic abuse (Bjørnholt & Hjemdal, 2018; Donovan & Barnes, 2021; Walby & Towers, 2018). The documentation of the links between animal abuse and other forms of domestic abuse also substantiates appeals to include animal abuse in the societal responses to domestic abuse. This has relevance for practice, both within child welfare, animal welfare, and support services for victims of domestic abuse. Families enduring domestic abuse inflict a high-stress and unpredictable environment upon children, and the exposure to animal abuse may be particularly traumatic for children who seek to a cherished companion animal for security and attachment (McDonald et al., 2015, 2019). Children’s exposure to animal abuse is a potential adverse experience that can contribute to toxic stress and long-term related health outcomes. Thus, asking about animal abuse can be an important tool for child welfare workers, health professionals, and forensic interviewers (Boat, 2014; Risley-Curtiss et al., 2010).

### Knowledge Gaps and Future Directions

This study investigated the occurrence of companion animal abuse in a representative sample of adolescents in Norway. There is also a need for similar studies with representative samples of the adult population. The revealed differences in the strengths of associations between animal abuse and psychological abuse and less severe physical abuse on one hand, and severe physical abuse on the other hand, should be further explored, both in quantitative and qualitative studies. The role of animal abuse as part of psychological abuse and coercive control of children also warrants further investigation.

This study did not include questions of children’s own involvement in animal abuse. This is also an important topic for future research, adding to the growing literature on children’s role in animal abuse (e.g., Connor et al., 2021; Currie, 2006; Henry, 2004; 2006; McEwen et al., 2014; Signal et al., 2013) and in domestic abuse, including coercive control (e.g., Dragiewicz et al., 2021). More knowledge about the different forms of animal abuse that occur in the context of domestic abuse is also needed to improve the identification and response to animal abuse.

## Conclusions

Our study presents unique data on the occurrence of animal abuse in the family as reported by a large and representative sample of 12 to 16-year-old youth in Norway. It confirms the links between animal abuse and child abuse, as indicated in previous research based on less representative samples.

In contrast to previous studies, animal abuse was most strongly associated with psychological abuse and the less severe forms of physical abuse experienced by the children in this study. This points towards an understanding of domestic abuse that emphasizes psychological abuse and coercive control as an ongoing pattern of abuse, rather than as single incidents of physical abuse. However, more severe forms of physical abuse and child sexual abuse also overlapped with animal abuse, albeit to a lesser degree. Apart from the presence of other forms of domestic abuse, the strongest risk factors for animal abuse in families with children and companion animals, were low socioeconomic status and parental risk factors. Both children and animals are more at risk of abuse as the number of risk factors increase.

We conclude that companion animal abuse co-occurs with other forms of domestic abuse and may be considered a part of the repertoire of domestic abuse that children are exposed to. Our study therefore confirms that non-human household members also may be at risk of abuse in families in which domestic abuse against humans occurs, and vice versa.
